# Gender Matters: A Multidimensional Approach to Optimizing Cardiovascular Health in Women

**DOI:** 10.7759/cureus.61810

**Published:** 2024-06-06

**Authors:** Tanya Sinha, Danyal Bakht, Syed Faqeer Hussain Bokhari, Maaz Amir, Rida Fatima, Kinza Bakht, Aisha Amir, Asma Aslam, Muzaffer Hussain, Tamseer Tariq

**Affiliations:** 1 Internal Medicine, Tribhuvan University, Kathmandu, NPL; 2 Medicine and Surgery, Mayo Hospital, Lahore, PAK; 3 Surgery, King Edward Medical University, Lahore, PAK; 4 Medicine and Surgery, King Edward Medical University, Lahore, PAK; 5 Medicine and Surgery, Fatima Jinnah Medical University, Lahore, PAK; 6 Internal Medicine, Sheikh Zayed Medical College and Hospital, Rahim Yar Khan, PAK; 7 Medicine and Surgery, Karachi Medical and Dental College, Karachi, PAK; 8 Surgery, Karachi Medical and Dental College, Karachi, PAK

**Keywords:** coronary heart disease, gender comparison, atherosclerosis, review, cardiovascular, women

## Abstract

Cardiovascular diseases remain a leading cause of mortality among women, yet they are often underestimated and insufficiently addressed. This narrative review delves into the gender disparities in cardiovascular health, underscoring the critical importance of recognizing and addressing the unique challenges women face. The article explores the pathophysiological differences between men and women, highlighting the role of hormonal factors, such as estrogen and menopause, in conferring cardioprotection or increasing risk. It examines the complexities of diagnosis and assessment, including differences in symptom presentation, diagnostic accuracy, and the challenges of interpreting non-invasive testing in women. The review also highlights the need for tailored risk assessment and prevention strategies, incorporating sex-specific conditions and pregnancy-related factors. It emphasizes the importance of lifestyle modifications and interventions, as well as the potential benefits of personalized treatment approaches, considering gender-specific variations in medication responses and cardiac interventions. Furthermore, the article sheds light on the impact of psychosocial and sociocultural factors, such as gender norms, mental health considerations, and access to healthcare, on women's cardiovascular health. It also addresses the significant gaps and challenges in research, including the historical underrepresentation of women in clinical trials and the lack of sex- and gender-sensitive studies. Finally, the review advocates for a multidisciplinary approach, involving patient-centered care, shared decision-making, and collaboration among policymakers, stakeholders, and healthcare systems. This comprehensive strategy aims to enhance awareness, prevention, diagnosis, and treatment of cardiovascular disease in women, ultimately improving health outcomes and reducing the burden of this often overlooked epidemic.

## Introduction and background

Heart disease in women is a significant and often underestimated health concern. Since 1984, the annual mortality rate due to cardiovascular disease among women has exceeded that of men, resulting in over 450,000 female deaths annually, with 250,000 attributed to coronary artery disease [1]. Despite these stark statistics, a prevailing misconception exists among women that breast cancer poses a more imminent threat. This misperception has dire consequences, particularly as women tend to develop heart disease approximately 10 years later than men but face poorer outcomes post-heart attack, in part due to underrecognized symptoms. Shockingly, an estimated 35% of heart attacks in women go unnoticed or unreported [1].

Despite the common association of coronary heart disease (CHD) with men and discussions often leaning towards breast cancer in women's health, the reality is vastly different. CHD takes the top spot among female mortality worldwide, second only to lower respiratory infections, neonatal disorders, diarrhea, and malaria in low-income nations [2]. Additionally, women are at higher risk for comorbid conditions such as diabetes and hypertension due to increased age, further complicating their cardiovascular health. For women, warning signs of myocardial ischemia encompass more than just chest tightness or discomfort; they also frequently include symptoms like nausea and dizziness. Furthermore, women may experience breathlessness, perspiration, a sensation of heart fluttering, and chest fullness as indicators of this condition [1].

The increasing recognition of both traditional and gender-specific risk factors has deepened our understanding of the mechanisms behind these concerning outcomes for women. Yet, a significant gender gap persists in preventive treatments. Women receive recommended therapies like lipid-lowering medication, aspirin, and lifestyle adjustments less frequently than men with similar cardiovascular risk profiles. Even when prescribed, treatments for women tend to be less aggressive and may not attain optimal results. Additionally, women's engagement in cardiac rehabilitation is markedly lower, primarily due to healthcare providers' inadequate referrals [3].

This narrative review thoroughly explores the often-underestimated problem of heart disease among women. It addresses gender disparities in cardiovascular health, divergences in the way men and women experience heart-related issues, the complexities of diagnosis, assessing risks, and implementing personalized treatment strategies. Furthermore, the article sheds light on the impact of psychosocial and sociocultural factors, emphasizing the imperative to include more women in clinical trials. The narrative promotes a multidisciplinary approach to cater to gender-specific cardiovascular health needs, ultimately striving to enhance awareness, prevention, diagnosis, and treatment in the field of women's cardiology.

## Review

Gender disparities in cardiovascular health

Gender disparities in cardiovascular health are a notable concern, given that cardiovascular disease (CVD) remains the leading cause of death among women. Women account for 52.6% of all CVD-related deaths. Recent statistics underscore the severity of this issue, revealing a significant number of female CVD-related deaths in the United States. While substantial progress has been made in reducing heart disease mortality in recent decades for both genders, this positive trend has stagnated, particularly among younger women. Since 1984, the number of cardiovascular disease (CVD) deaths in women has consistently exceeded those in men [3,4]. One significant aspect of gender-specific risk factors involves the complex interaction of classical risk factors that are shared by both genders. The relative importance and impact of these risk factors vary between genders. For example, in younger women, smoking has a disproportionately negative effect on cardiovascular health, increasing the risk of acute myocardial infarction [5,6]. Changes in body weight and fat distribution due to menopause contribute to a higher prevalence of obesity, type 2 diabetes, and metabolic syndrome in women compared to men [5,7]. As women age, systolic blood pressure rises more steeply compared to men, potentially influenced by hormonal fluctuations. Moreover, elevated cholesterol levels become more pronounced after menopause, highlighting the importance of monitoring lipid profiles during this life stage. Research has demonstrated that hormonal imbalances in premenopausal women are linked to a heightened risk of atherosclerosis and coronary heart disease (CHD) occurrences. Females who have experienced hypertensive disorders during pregnancy face an elevated risk of developing hypertension and experiencing premature cardiovascular diseases in the future [5].

In addition to physiological factors, the perception of heart disease in women plays a significant role. Many women do not view heart disease as a substantial health concern and often lack awareness of their risk. Surprisingly, age appears to substantially influence knowledge more than ethnicity, emphasizing the need for educational initiatives aimed at younger women. Furthermore, the impact of societal norms and prevailing perceptions regarding age and gender significantly contributes to low awareness among young women, potentially hindering the adoption of heart-healthy behaviors. Addressing these awareness and perception gaps requires more effective communication between healthcare providers and patients [8,9].

Pathophysiological differences

Significant pathophysiological distinctions in cardiovascular health between women and men have far-reaching implications for various aspects of cardiovascular well-being. The misperception that women inherently possess protection against cardiovascular disease has led to the underestimation of their risk. This, in turn, has resulted in variations in clinical presentation and less aggressive treatment strategies. These misconceptions have given rise to less aggressive treatment strategies and lower representation of women in clinical trials, hampering our understanding of women's unique aspects of cardiovascular health [5]. Numerous hormonal factors contribute to these pathophysiological differences. Notably, endogenous female sex hormones, primarily estrogens, play a pivotal role in conferring cardioprotection. Estrogens engage in multiple mechanisms that benefit heart health, including elevating high-density lipoprotein (HDL) levels, lowering low-density lipoprotein (LDL) levels, and stimulating the release of vasodilators like nitric oxide and prostacyclin from vessel walls. This leads to reduced blood pressure and diminished platelet aggregation [10,11]. However, in post-menopause, there is a pronounced increase in blood pressure, often exceeding levels observed in men. Concurrently, during the menopausal transition, there is a notable increase in the prevalence of metabolic syndrome, increased body weight, dyslipidemia, hyperinsulinemia, and hypertension [11]. This hormonal shift and its repercussions for heart health underscore the critical importance of comprehending the role of menopause in cardiovascular health.

The transition through menopause, characterized by hormonal changes and modifications in cardiovascular risk factors, serves to underscore the intricacy of the pathophysiological disparities in heart health between women and men. While premenopausal women experience relative protection against cardiovascular disease compared to age-matched men, this safeguard diminishes after menopause [3]. This observation has led to the hypothesis that menopause plays a contributory role in the heightened risk of coronary heart disease among women. Longitudinal investigations conducted during the menopausal transition have documented distinct patterns of sex hormone alterations, adverse modifications in body composition, lipids, lipoproteins, and measures of vascular health. These changes collectively elevate the risk of developing cardiovascular disease in women post-menopause [12]. This emphasis on the menopausal transition as a pivotal period for cardiovascular risk underlines the significance of monitoring and addressing these pathophysiological differences.

Another area of pathophysiological divergence pertains to distinct cardiac conditions that predominantly affect women. Microvascular coronary dysfunction (MCD), characterized by restricted coronary flow reserve and/or malfunctioning coronary endothelium, represents the primary underlying cause of ischemia in women presenting with the trio of persistent chest pain, unobstructed coronary arteries, and evidence of ischemia during stress testing. This condition translates into a greater symptom burden, heightened functional disability, and a higher incidence of non-obstructive coronary artery disease in comparison to men [13]. Another cardiac ailment that predominantly afflicts women is stress cardiomyopathy, an ailment provoked by intense emotional or physical stress characterized by transient apical systolic dysfunction with left ventricular ballooning. Stress cardiomyopathy primarily afflicts postmenopausal women, underscoring the substantial influence of hormonal and emotional factors on women's cardiac health [14].

Diagnosis and assessment

The diagnosis and assessment of heart disease, particularly in women, present unique challenges and complexities. One significant aspect of this challenge is the gender bias in cardiac symptom presentation. It has been observed that women and men tend to describe their angina symptoms differently, with men often using the term "chest pain" while women describe sensations such as "heaviness." This distinction in symptom description may lead to delays in women reporting their symptoms, contributing to later diagnosis and treatment [15]. Additionally, women may experience less common signs of angina, such as shortness of breath, fatigue, sweating, and weakness, which can be labeled as "atypical angina" [16,17]. Recognizing the possibility of angina, regardless of symptom description, is crucial in ensuring timely diagnosis and intervention.

The challenges in diagnosing heart disease in women go beyond symptom presentation. Women are often evaluated for coronary artery disease (CAD) much later than men, causing delays in diagnosis. In comparison, most women present with the same CAD symptoms as men, and a significant number experience atypical symptoms. This complicates the diagnostic process, with some women diagnosed with myocardial infarction not reporting chest pain as their primary symptom [18]. Moreover, when women with acute coronary syndromes (ACS) undergo cardiac catheterization, a higher proportion do not have significant obstructive CAD. Yet, their prognosis is worse, making diagnosis more complex [19].

The interpretation of noninvasive diagnostic testing is also less reliable in women, particularly in the age group of under 55 years, when CAD prevalence is relatively low. This highlights the need for gender-specific diagnostic tools and guidelines. Traditional diagnostic methods like the exercise electrocardiogram may have lower accuracy in women, partly due to hormonal variations. Pharmacological stress testing, stress echocardiography, and computed tomography coronary angiography are mentioned as alternative diagnostic techniques with better accuracy for CAD in women. However, factors like breast tissue and obesity can still affect the accuracy of some diagnostic methods in women [14].

Risk assessment and prevention

CVD prevention in women presents a distinctive array of challenges and considerations within the realm of medical research. Historically, women have exhibited a lower likelihood than men of having their cardiovascular risk factors identified and managed at an early stage of life [2,20]. A fundamental element in assessing risk among women involves the recognition of sex-specific conditions that heighten their susceptibility to CVD. These conditions encompass a history of premature menopause and various pregnancy-related factors, such as gestational hypertension, preeclampsia (hypertensive disorders of pregnancy), gestational diabetes, and giving birth to small-for-gestational-age infants. These factors, denoted as "risk-enhancing factors," independently classify women as being at an increased prospective risk of atherosclerotic CVD. However, there is often a notable lack of awareness, both among patients and healthcare providers, concerning the role these pregnancy-related factors play in the assessment of CVD risk. This deficiency leads to inadequate follow-up and monitoring, particularly among young women and underserved populations [21].

To tackle these challenges, the implementation of preventive measures tailored specifically to women's needs is imperative. The utilization of statins for primary CVD prevention, as exemplified by trials such as JUPITER, holds value for both men and women [22]. However, it is crucial to provide comprehensive counseling to women of childbearing age regarding contraceptive practices when undergoing statin therapy due to the paucity of safety data about statin usage during pregnancy. Similarly, aspirin therapy, well-established for secondary CVD prevention, has demonstrated efficacy in reducing the risk of significant cardiovascular events and ischemic stroke in women, particularly those aged 65 and above [23].

Lifestyle modifications and interventions remain foundational components in the prevention and management of CVD. Engaging in increased physical activity, adhering to appropriate nutritional practices, managing body weight, abstaining from tobacco use, and addressing stressors are universally applicable strategies [24]. These strategies not only serve to lower the risk of CVD but also contribute to an overall enhancement of quality of life. A multidisciplinary approach that encompasses dietary adjustments, exercise regimens, and behavioral modifications can effectively target and modify multiple risk factors, thereby diminishing the risk of heart disease, enhancing survival rates, and elevating the quality of life among individuals, regardless of their gender [25]. In this regard, healthcare professionals are bestowed with a pivotal role in identifying and promoting these favorable lifestyle measures as central strategies for mitigating CVD risk and improving comprehensive cardiovascular health [24].

Treatment approaches

Treatment approaches in the context of cardiovascular health encompass a nuanced understanding of gender-specific differences in medication responses. It is evident from a retrospective analysis of cardiovascular drug trials that women and men exhibit variations in drug efficacy and adverse effects. This divergence includes higher mortality rates among women taking digoxin for heart failure, increased risk of torsade de pointes arrhythmia with QT-prolonging drugs, and a higher incidence of cough associated with angiotensin-converting enzyme (ACE) inhibitors. However, it is important to note that while trends towards a greater benefit for women have been observed in some cases for specific drugs like amlodipine, ramipril, and eplerenone, further research is needed to establish the statistical significance of these differences. Moreover, some drugs, such as metoprolol and verapamil, require precise dose adaptations for women due to variations in pharmacokinetics and pharmacodynamics, highlighting the necessity of gender-specific considerations in cardiovascular drug therapy [26].

Cardiac interventions and procedures tailored to women are a pivotal aspect of improving cardiovascular care. Women's unique physiological characteristics, influenced by factors like hormonal variations during the menstrual cycle, menopause, and pregnancy, necessitate tailored approaches to cardiac treatment. While current guidelines for cardiovascular disease prevention provide valuable frameworks, there is a growing recognition of the need to develop individualized therapeutic strategies that account for gender-specific differences in drug responses [27]. This is vital to enhance the safety and efficacy of cardiovascular drugs for women.

Pregnancy-related cardiac care has gained national attention due to the prominent role of cardiac conditions in maternal morbidity and mortality. As the number of women with congenital heart disease reaching childbearing age grows, it's crucial to address contraceptive options in collaboration with cardiologists [28]. For those desiring pregnancy, referral to a pregnancy heart team is recommended. American College of Obstetricians and Gynecologists (ACOG) advises delivering in pregnancy heart centers for moderate- to high-risk cardiac patients (modified WHO risk classes III and IV) as outcomes significantly improve [29]. Coordination within multidisciplinary teams and personalized care pathways are essential. While cesarean delivery rates are higher, vaginal delivery is preferred when possible, with proper neuraxial anesthesia. In some cases, cardiac interventions are necessary, with percutaneous procedures generally posing lower risks. Cardiopulmonary bypass surgery may require adjusted perfusion protocols. When severe cardiovascular or respiratory failure occurs, extracorporeal membrane oxygenation (ECMO) serves as a life-saving measure with a survival rate of up to 70%, subject to the underlying disease etiology [30].

Psychosocial and sociocultural factors

Psychosocial and sociocultural factors play a significant role in shaping heart health, with a particular focus on the influence of gender social norms. Gender social norms, often unspoken yet deeply ingrained, have a profound impact on how individuals enact their gender and interact with others. These norms contribute to a range of cardiovascular health outcomes, encompassing healthcare disparities, health behaviors, and broader determinants of health. Biased gender-related social norms could be linked to behaviors that harm cardiovascular health in both genders, potentially raising the burden of cardiovascular disease and diminishing life expectancy [31]. The oversight of gender's significance in shaping cardiovascular health is regrettable, despite its crucial role. It's evident that CVD experiences and outcomes vary by biological sex. However, the tendency to blur the lines between sex and gender has fostered the misconception that both are immutable, with limited opportunities for intervention [32]. Gender norms can shape behaviors and social relations, impacting factors such as stress and smoking, which are risk factors for cardiovascular disease [31].

Mental health considerations, especially in women with heart disease, are a critical aspect of heart health. Women are more vulnerable to common mental disorders such as depression and anxiety across their life course [33]. This vulnerability has implications for the prevention, identification, and treatment of both mental health disorders and coronary heart disease in women. Stress, both job-related and marital, has been associated with depression symptoms in women, intensifying the risks of coronary heart disease. Anxiety, with its physical symptoms and muscle tension, has also been linked to an increased risk of coronary heart disease in women [34].

Access to healthcare and disparities in treatment further compound these issues. Women often experience inadequate treatment in comparison to men, which is evident in the case of ST elevation myocardial infarction (STEMI). These disparities extend to various aspects of heart health, from the use of guideline-directed therapies to revascularization and cardiac rehabilitation. Delays in acute cardiovascular care for women can be attributed to a range of factors, including patients' longer intervals between symptom onset and presentation, which, in turn, may result from the healthcare team's failure to recognize acute coronary syndromes in women due to gaps in symptom understanding and potential biases [35]. It's crucial to highlight that even among women with obstructive CAD and typical symptoms, adherence to recommended guidelines for invasive and pharmacologic treatments isn't consistently observed, similar to what's seen in men [36].

Challenges and gaps in research

Research on heart disease in women faces several significant challenges and gaps that must be addressed to advance our understanding of cardiovascular health in this demographic. One of the most pressing issues is the historical underrepresentation of women in cardiovascular clinical trials. While studies like the Women's Health Study (WHS) and the Women's Health Initiative (WHI) have increased female participation, women continue to be inadequately represented, accounting for less than a third of participants in mixed-gender trials [23]. This gender disparity in research participation has significant implications for the development of effective therapeutic strategies for women.

Several factors contribute to the low enrollment of women in cardiovascular trials. First, there is a prevalent underestimation of cardiac risk in women, which can result in atypical cardiac disease presentation and, consequently, reduced referrals to cardiology practices where recruitment for cardiovascular clinical trials typically occurs [23]. Additionally, women tend to manifest cardiovascular diseases later in life, which can introduce an age-gender bias in the study enrollment process [37]. Other barriers include fear and distrust of the research enterprise, lack of knowledge about clinical trials, limited access to transportation, interference with work and family responsibilities, subject burden due to study participation, and financial costs [23]. All of these factors contribute to the underrepresentation of women in cardiovascular research. The lack of sex- and gender-sensitive studies in CVD research highlights a significant gap in the field of non-communicable disease research [3]. Women's cardiovascular health has been historically sidelined, with a common misconception that CVD primarily affects men or that both genders are equally susceptible. However, research has consistently demonstrated notable heterogeneity in CVD manifestation, risk-factor burden, and disease prognosis between men and women. This heterogeneity arises from both biological factors (sex) and various social determinants (gender identity) [38]. Furthermore, there is a substantial lack of awareness among women regarding the threat of cardiovascular disease. Many women mistakenly identify breast cancer as the leading cause of death in their demographic, leading to delays in seeking medical advice for heart-related issues [39-41]. Physicians also tend to underestimate women's cardiovascular risk, potentially resulting in underdiagnosis, inappropriate treatment decisions, or untreated conditions [38].

To address these gender disparities in cardiovascular research and healthcare, there is an urgent need for sustained efforts and commitments to develop tailored strategic plans for the prevention and management of heart disease in women. Policymakers must design and evaluate strategies to increase awareness, enhance research, and optimize prevention, diagnosis, and treatment for women [42]. This critical step is essential to bridge the gaps and challenges in our understanding of heart disease in women and ultimately improve cardiovascular health outcomes in this population.

Multidisciplinary approaches

The implementation of multidisciplinary strategies is of paramount importance in addressing heart diseases and enhancing heart health in women. These approaches are pivotal in achieving improved cardiovascular outcomes and a reduction in maternal morbidity and mortality. Integrated models of cardiology and obstetric care have been suggested to tackle maternal mortality attributed to CVD in the US, with a study revealing that nearly half of serious cardiac events in patients with pre-existing heart conditions during pregnancy could be prevented, often due to provider-related factors. Cardio-obstetrics, involving a collaborative team approach, including maternal-fetal medicine, cardiology, anesthesiology, neonatology, nursing, social work, and pharmacy, is crucial for addressing maternal CVD outcomes. The American Heart Association emphasizes its role in reducing maternal morbidity and mortality throughout pregnancy [43].

Patient-centered care (PCC) plays a pivotal role in women's cardiac health, emphasizing a collaborative partnership between healthcare practitioners, patients, and their families to meet individual care needs, values, and preferences [44]. This approach fosters empathy, respect, effective communication, and shared decision-making. PCC in women's health results in improved patient knowledge, enhanced self-care skills, increased satisfaction, better medication adherence, enhanced quality of life, and reduced hospital admissions and readmissions. Shared decision-making, a central component of PCC, facilitates patient engagement, enabling individuals to actively participate in their healthcare decisions [45]. This approach is evolving globally, reflecting varying attitudes and practices, making PCC an essential and adaptable model in women's health.

**Figure 1:**
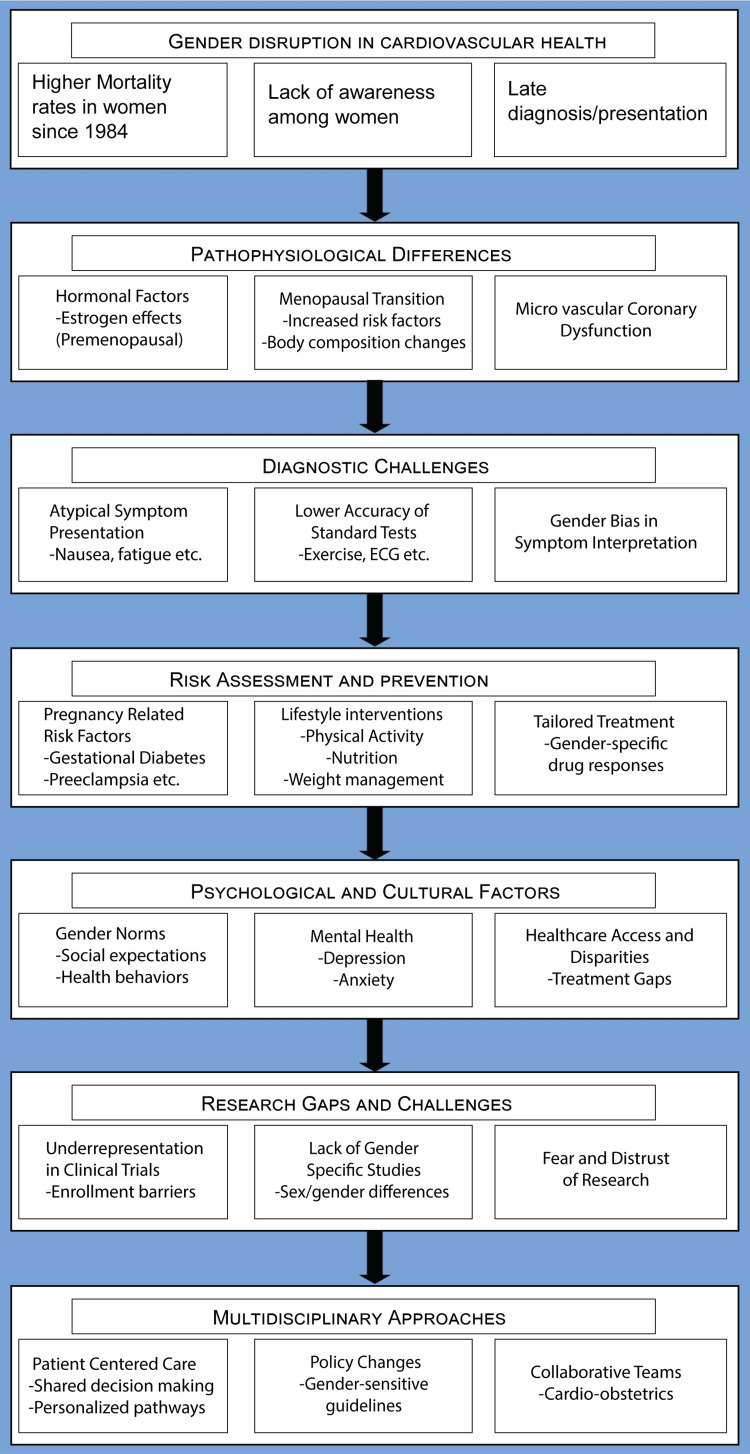
Flowchart navigating the essence of this article for clarity and simplicity. This image has been generated by the authors.

To optimize the cardiac health of women, it is imperative to adopt a comprehensive and gender-specific strategy. Policymakers need to not only recognize but also actively promote and allocate resources to address sex- and gender-specific issues across the entire spectrum of cardiovascular disease (CVD), encompassing prevention, risk assessment, diagnosis, treatment, and rehabilitation [46]. A fundamental step in this process involves conducting a needs assessment to thoroughly evaluate national healthcare requirements. The multidimensional approach, as advocated by public health initiatives, necessitates the collaborative efforts of policymakers, stakeholders, researchers, and healthcare systems. Such collaboration is indispensable for formulating evidence-based and cost-effective decisions that cater to individual needs. Key components of this multifaceted strategy encompass the integration of sex- and gender-based guidelines in CVD prevention and management, the provision of patient-centered care designed to address diverse cultural, ethnic, spiritual, and social determinants, and the establishment of multidisciplinary healthcare teams specifically dedicated to women. This team-based approach has the potential to facilitate comprehensive evaluation and treatment, ultimately optimizing the patient experience [38]. The gist of this review is summarized in the following image (Figure 1).

## Conclusions

Cardiovascular disease in women is a complex and multifaceted issue that demands urgent attention and comprehensive efforts. It remains the leading cause of death among women worldwide despite persistent misperceptions and underestimations of the disease's impact. Gender disparities in cardiovascular health have deep-rooted causes, spanning from societal norms and expectations to hormonal and physiological differences. Addressing these disparities and fostering a better understanding of gender-specific aspects of heart disease is essential to improving prevention, diagnosis, and treatment. This narrative review underscores the urgency of recognizing and rectifying gender disparities in cardiovascular health and research. Women's unique risk factors and pathophysiological differences require tailored approaches, both in research and clinical practice. The complexities of symptom presentation, diagnostic accuracy, and treatment responses in women make it evident that the one-size-fits-all approach to cardiovascular care is insufficient. By incorporating gender-sensitive guidelines, promoting women's participation in clinical trials, and implementing multidisciplinary approaches to heart disease, we can take significant steps toward reducing the burden of cardiovascular disease in women. Healthcare providers, policymakers, and researchers need to collaborate and commit to addressing the challenges and gaps in cardiovascular research, prevention, and treatment. Such efforts are pivotal to ensure that women receive the care and support they need to achieve and maintain optimal cardiovascular health.

## References

[1] Giardina EG (2000). Heart disease in women. Int J Fertil Womens Med.

[2] Woodward M (2019). Cardiovascular disease and the female disadvantage. Int J Environ Res Public Health.

[3] Garcia M, Mulvagh SL, Merz CN, Buring JE, Manson JE (2016). Cardiovascular disease in women: clinical perspectives. Circ Res.

[4] Zhang Y (2010). Cardiovascular diseases in American women. Nutr Metab Cardiovasc Dis.

[5] Maas AH, Appelman YE (2010). Gender differences in coronary heart disease. Neth Heart J.

[6] Vanhoutte PM, Shimokawa H, Tang EH, Feletou M (2009). Endothelial dysfunction and vascular disease. Acta Physiol (Oxf).

[7] Kip KE, Marroquin OC, Kelley DE (2004). Clinical importance of obesity versus the metabolic syndrome in cardiovascular risk in women: a report from the Women's Ischemia Syndrome Evaluation (WISE) study. Circulation.

[8] Gooding HC, Brown CA, Revette AC (2020). Young women's perceptions of heart disease risk. J Adolesc Health.

[9] Mosca L, Jones WK, King KB, Ouyang P, Redberg RF, Hill MN (2000). Awareness, perception, and knowledge of heart disease risk and prevention among women in the United States. American Heart Association Women's Heart Disease and Stroke Campaign Task Force. Arch Fam Med.

[10] Barrett-Connor E, Bush TL (1991). Estrogen and coronary heart disease in women. JAMA.

[11] Pérez-López FR, Larrad-Mur L, Kallen A, Chedraui P, Taylor HS (2010). Gender differences in cardiovascular disease: hormonal and biochemical influences. Reprod Sci.

[12] El Khoudary SR, Aggarwal B, Beckie TM (2020). Menopause transition and cardiovascular disease risk: implications for timing of early prevention: a scientific statement from the American Heart Association. Circulation.

[13] Kothawade K, Bairey Merz CN (2011). Microvascular coronary dysfunction in women: pathophysiology, diagnosis, and management. Curr Probl Cardiol.

[14] Keteepe-Arachi T, Sharma S (2017). Cardiovascular disease in women: understanding symptoms and risk factors. Eur Cardiol.

[15] Desai S, Munshi A, Munshi D (2021). Gender bias in cardiovascular disease prevention, detection, and management, with specific reference to coronary artery disease. J Midlife Health.

[16] Johnson BD, Kelsey SE, Merz CNB (2004). Clinical risk assessment in women. Coronary Disease in Women.

[17] Douglas PS, Ginsburg GS (1996). The evaluation of chest pain in women. N Engl J Med.

[18] Brewer LC, Svatikova A, Mulvagh SL (2015). The challenges of prevention, diagnosis and treatment of ischemic heart disease in women. Cardiovasc Drugs Ther.

[19] Johnson BD, Shaw LJ, Pepine CJ (2006). Persistent chest pain predicts cardiovascular events in women without obstructive coronary artery disease: results from the NIH-NHLBI-sponsored Women's Ischaemia Syndrome Evaluation (WISE) study. Eur Heart J.

[20] Khamis RY, Ammari T, Mikhail GW (2016). Gender differences in coronary heart disease. Heart.

[21] Marschner S, Mukherjee S, Watts M (2023). Prevention of cardiovascular disease in women with pregnancy-related risk factors: a prospective women's heart clinic study. J Am Heart Assoc.

[22] Ridker PM, Danielson E, Fonseca FA (2008). Rosuvastatin to prevent vascular events in men and women with elevated C-reactive protein. N Engl J Med.

[23] Saeed A, Kampangkaew J, Nambi V (2017). Prevention of cardiovascular disease in women. Methodist Debakey Cardiovasc J.

[24] Rippe JM (2019). Lifestyle strategies for risk factor reduction, prevention, and treatment of cardiovascular disease. Am J Lifestyle Med.

[25] Khouja JH, Al Jasir B, Bargawi AA, Kutbi M (2020). Lifestyle Intervention for cardiovascular disease risk factors in Jeddah, Saudi Arabia. Cureus.

[26] Seeland U, Regitz-Zagrosek V (2012). Sex and gender differences in cardiovascular drug therapy. Handb Exp Pharmacol.

[27] Tamargo J, Rosano G, Walther T (2017). Gender differences in the effects of cardiovascular drugs. Eur Heart J Cardiovasc Pharmacother.

[28] (2020). Gynecologic considerations for adolescents and young women with cardiac conditions: ACOG Committee Opinion, Number 813. Obstet Gynecol.

[29] (2019). ACOG practice bulletin no. 212: pregnancy and heart disease. Obstet Gynecol.

[30] Hussey H, Hussey P, Meng ML (2021). Peripartum considerations for women with cardiac disease. Curr Opin Anaesthesiol.

[31] Lyell I, Khan SS, Limmer M, O'Flaherty M, Head A (2023). Association between gender social norms and cardiovascular disease mortality and life expectancy: an ecological study. BMJ Open.

[32] O'Neil A, Scovelle AJ, Milner AJ, Kavanagh A (2018). Gender/sex as a social determinant of cardiovascular risk. Circulation.

[33] O'Neil A, Russell JD, Murphy B (2021). How does mental health impact women's heart health?. Heart Lung Circ.

[34] Khayyam-Nekouei Z, Neshatdoost H, Yousefy A, Sadeghi M, Manshaee G (2013). Psychological factors and coronary heart disease. ARYA Atheroscler.

[35] Gulati M, Hendry C, Parapid B, Mulvagh SL (2021). Why we need specialised centres for women's hearts: changing the face of cardiovascular care for women. Eur Cardiol.

[36] Vasiljevic-Pokrajcic Z, Krljanac G, Lasica R (2021). Gender disparities on access to care and coronary disease management. Curr Pharm Des.

[37] Lee PY, Alexander KP, Hammill BG, Pasquali SK, Peterson ED (2001). Representation of elderly persons and women in published randomized trials of acute coronary syndromes. JAMA.

[38] Kouvari M, Souliotis K, Yannakoulia M, Panagiotakos DB (2020). Cardiovascular diseases in women: policies and practices around the globe to achieve gender equity in cardiac health. Risk Manag Healthc Policy.

[39] Bairey Merz CN, Andersen H, Sprague E (2017). Knowledge, attitudes, and beliefs regarding cardiovascular disease in women: the women's heart alliance. J Am Coll Cardiol.

[40] Webster R, Heeley E (2010). Perceptions of risk: understanding cardiovascular disease. Risk Manag Healthc Policy.

[41] Kouvari M, Yannakoulia M, Souliotis K, Panagiotakos DB (2018). Challenges in sex- and gender-centered prevention and management of cardiovascular disease: implications of genetic, metabolic, and environmental paths. Angiology.

[42] Norton R (2016). Women's health: a new global agenda. Womens Health (Lond).

[43] Magun E, DeFilippis EM, Noble S, LaSala A, Waksmonski C, D'Alton ME, Haythe J (2020). Cardiovascular care for pregnant women with cardiovascular disease. J Am Coll Cardiol.

[44] Gagliardi AR, Green C, Dunn S, Grace SL, Khanlou N, Stewart DE (2019). How do and could clinical guidelines support patient-centred care for women: content analysis of guidelines. PLoS One.

[45] Marques MD, Pires R, Perdigão M, Sousa L, Fonseca C, Pinho LG, Lopes M (2021). Patient-centered care for patients with cardiometabolic diseases: an integrative review. J Pers Med.

[46] Karwalajtys T, Kaczorowski J (2010). An integrated approach to preventing cardiovascular disease: community-based approaches, health system initiatives, and public health policy. Risk Manag Healthc Policy.

